# Complete genome sequence of a *Desulfovibrio legallii* clinical isolate, GTC18478, from Japan

**DOI:** 10.1128/mra.00676-25

**Published:** 2025-09-11

**Authors:** Masahiro Hayashi, Shinichiro Kobayashi, Jun Yonetamari, Yoshinori Muto, Jun Suzuki, Kaori Tanaka

**Affiliations:** 1 Institute for Glyco-core Research iGCORE, Gifu University 12785, Gifu City, Gifu, Japan; 2 Division of Anaerobe Research, Life Science Research Center, Gifu University 201111, Gifu City, Gifu, Japan; 3 Gifu University Center for Conservation of Microbial Genetic Resource 12785, Gifu, Japan; 4 Department of Infectious Disease, Gifu Prefectural General Medical Center 68266, Gifu, Japan; 5 Division of Clinical Laboratory, Gifu University Hospital 476117, Gifu, Japan; 6 United Graduate School of Drug Discovery and Medical Information Sciences, Gifu University 520344 , Gifu City, Gifu, Japan; 7 Center for One Medicine Innovative Translational Research (COMIT), Gifu University 12785, Gifu, Japan; University of Maryland School of Medicine, Baltimore, Maryland, USA

**Keywords:** *Desulfovibrio legallii*, genome, anaerobes

## Abstract

We report the complete genome sequence of a clinical isolate of *Desulfovibrio legallii* obtained from a human patient in Japan. The genome consists of a 2,750,737-bp circular chromosome.

## ANNOUNCEMENT

*Desulfovibrio* species are non-spore-forming, nonfermentative, sulfate-reducing, anaerobic, Gram-negative bacilli. Most are motile, with polar flagella, and curved or spiral in morphology. These species are commonly found in various environments and can cause human infections (1, 2). *Desulfovibrio legallii* strain GTC18478 was identified in a human clinical specimen in Gifu, Japan, in 2024 (3). The strain was isolated from an anaerobic blood culture and cultured on Brucella HK agar with 5% laked sheep blood at 37°C under anaerobic conditions (82% N_2_, 10% CO_2_, and 8% H_2_) for 48 h. The strain could not be identified by matrix-assisted laser desorption/ionization time-of-flight mass spectrometry (MALDI-TOF MS) (Bruker, Germany). It was stored at –80°C until use.

 Ethical approval for the present study was not necessary as no patient was involved. The genome was sequenced using a PromethION 2i platform (Oxford Nanopore Technologies [ONT], UK) for long-read sequencing and the NovaSeq 6000 system (Illumina, USA) for short-read sequencing, as previously described (4, 5). For DNA extraction, the strain was cultured once under the same conditions as those used for isolation as described above. After incubation, colonies were scraped, suspended in Tris-EDTA buffer, and pelleted through centrifugation. Genomic DNA was extracted from the bacterial pellet using NucleoBond HMW DNA (Macherey-Nagel, Japan). The long-read sequencing library was constructed using a Native Barcode Sequencing Kit (SQK-NBD114-24; ONT), without shearing, and sequenced using a FLO-PRO114M flow cell. MinkNOW v.24.06.15 was used for base calling, and the raw reads were trimmed and quality-filtered using NanoFilt v.2.7.1 (6) with the parameters “-l 1000 -q 10 -headcrop 50.” The short-read sequencing library was constructed using a DNA Prep (M) Tagmentation kit (Illumina) and sequenced using the NovaSeq 6000 platform, generating 2 × 151-bp paired-end reads. The raw reads were processed using fastp v.0.20.1 (7) with the parameters “-q 30 -n 20 -t 1 -T 1.” Short read quality was assessed using fastp v.0.20.1 (7). The mean read quality of the long reads was scored using NanoPlot v.1.32.1 (7). Short (>93.26% of bases > Q30 on average) and long (mean read quality of 20.0) reads were assembled using Flye v.2.9 (8). Assembly circularization and rotation were performed automatically using Flye v.2.9. The average nucleotide identity (ANI) was analyzed using PyANI v.0.2.12 (9). Default parameter settings were used for all software, unless otherwise specified.

Table 1 summarizes the genome information. Quality assessment was conducted using CheckM (v.1.2.2) (10), and genome statistics were computed using SeqKit (v.2.9.0) (11). CheckM results indicated that the genome was 99.11% complete, with 0.00% contamination. The DNA Databank of Japan (DDBJ) Fast Annotation and Submission Tool (12) predicted 2,310 coding sequences, nine ribosomal RNAs, 52 transfer RNAs, and 2 CRISPR sequences.

**TABLE 1:** Information on the complete genome sequence of *D. legallii* GTC18478 isolated from a human clinical specimen in Japan

Parameter	*D. legallii* GTC18478
NovaSeq 6000 sequencing^*a*^	
No. of reads	8,110,906 (7,583,878)^*e*^
Size (kb)	1,213,370
Average coverage (x)	441
DRA accession no.	DRR699507
ONT sequencing^*a*^	
No. of reads	135,516 (17,297)^*e*^
Size (kb)	1,611,496
Average read length (bp)	11,892
Average coverage (x)	586
N50	24,519
DRA accession No.	DRR699506
Assembly	
Assembly N50 (bp)^*c*^	2,750,737
Estimated genome completeness (%)^*d*^	99.11
Estimated genome contamination (%)^*d*^	0
Genome structure	one chromosome
DDBJ/GenBank accession No.	AP041046
Genome size (bp)	2,750,737
GC content (%)	
(chromosome/plasmid name)	62.5
No. of coding sequences^*b*^	2,310
No. of rRNAs^*b*^	9
No. of tRNAs^*b*^	52
No. of CRISPR sequences^*b*^	2

^*a*^ DRA, DDBJ Sequence Read Archive.

^*b*^ DFAST, DDBJ Fast Annotation and Submission Tool.

^*c*^ Determined with SeqKit v.2.3.0.

^*d*^ Determined with CheckM v.1.2.2.

^*e*^ The value in parentheses indicates the number of reads after quality filtering.

BLASTN analysis (13) of the 16S rRNA gene sequence against curated type strain database identified *D. legallii* H1^T^ (NCBI accession number NR_108301) as the closest match, with 99.65% identity. Whole-genome ANI between strain GTC18478 and *D. legallii* H1^T^ was 99.76%. Genome sequence-based species identification using GTDB-tK (v.2.4.0) confirmed the species as *D. legallii* (14).

## Data Availability

The genome sequence of *D. legallii* GTC18478 has been deposited in DDBJ (https://www.ddbj.nig.ac.jp/index.html) under the accession number AP041046. Raw sequence data for GTC18478 have been deposited in the DDBJ/Sequence Read Archive under the accession numbers DRR699506 and DRR699507.
